# Artificial urinary sphincter surgery in the UK: are we following the guidelines?

**DOI:** 10.1308/rcsann.2025.0104

**Published:** 2025-12-15

**Authors:** NR Bhatt, R Doherty, S Biers, C Harding, N Thiruchelvam, M Belal, A Sahai, H Hashim

**Affiliations:** ^1^ Newcastle upon Tyne Hospitals NHS Foundation Trust, UK; ^2^ Norfolk and Norwich University Hospitals NHS Foundation Trust, UK; ^3^ Cambridge University Hospitals NHS Foundation Trust, UK; ^4^ Translational and Clinical Research Institute, Newcastle University, UK; ^5^ Lewisham and Greenwich NHS Trust, UK; ^6^ Guy’s and St Thomas’ NHS Foundation Trust, UK; ^7^ North Bristol NHS Trust, UK; ^8^ University of Bristol, UK

**Keywords:** Male incontinence, Prostate surgery, Genitourinary sphincter, Artificial, Urinary stress incontinence

## Abstract

**Introduction:**

Artificial urinary sphincter (AUS) is a guideline-recommended treatment for male stress urinary incontinence. Despite its widespread use, it is thought that there is no standardisation in AUS practice. This study aims to report current AUS insertion practices in the UK and highlight any discrepancies.

**Methods:**

A REDCap survey was conducted under the British Association of Urological Surgeons (BAUS) Section of Female, Neurological, and Urodynamic Urology, and reported using the CHERRIES checklist. Outcomes were framed using the International Continence Society (ICS) document and recent BAUS consensus document.

**Results:**

The survey received 34 responses (response rate: 44–94%). Most respondents (80%) used video-urodynamics and patient-reported outcomes in patient workup. Loss of compliance on urodynamics was the most common contraindication, and detrusor overactivity was often treated before AUS surgery. Perioperative preparation and implantation techniques varied significantly from the ICS document, as did complication management.

**Conclusions:**

The reported variation may result from local or national influences, a lack of high-quality evidence and divergent surgical training. This variability impacts the heterogeneity of outcomes and their reporting. Future efforts should focus on adopting the new national consensus to standardise practice, improving training curricula, researching the effects of variability on surgical outcomes and enhancing the quality of evidence in this field.

## Introduction

**Figure 1:**
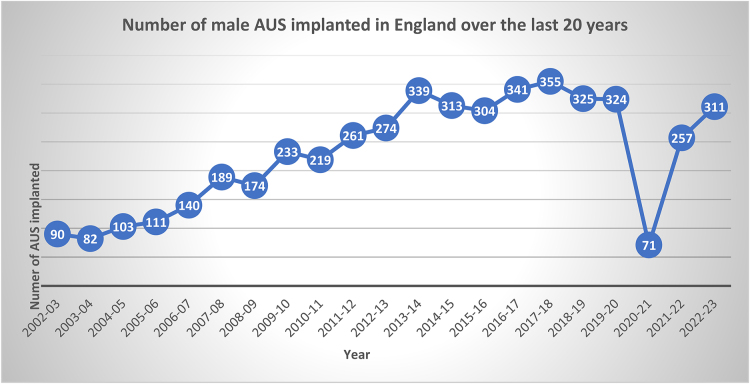
Number of male artificial urinary sphincter devices implanted in England over the past two decades (source: Health Episode Statistics data^3^)

Artificial urinary sphincter (AUS) is a guideline-recommended treatment option for male stress urinary incontinence (SUI). It was first introduced in 1973 and has undergone several design changes over subsequent decades. The AMS800 (American Medical Systems, Minnetonka, MN, USA) was introduced in 1983 and continues to be the most commonly used device.^1^ A report from 2016 indicated 11,500 AUS devices are placed annually worldwide.^2^ The number of AUS insertions globally has increased each year since its inception in 1973. Figure 1 shows the increasing trend in AUS implanted in England over the past two decades.^3^ The fall in AUS procedures in 2020 is probably due to the impact of the COVID pandemic on surgery for benign conditions. Hospital Episode Statistics in the United Kingdom (UK) report that 311 male AUS insertions were performed in the period 2022–2023 in England.^3^ Reports from the USA indicate only that 4% of urologists performing AUS surgery are considered high-volume surgeons (>20 insertions per year), with more than 90% of surgeons performing 5 or fewer cases per year (median 1 to 2) in most years.^4^ More than 90% of AUS implantations are reportedly performed for post-prostatectomy incontinence.^5^

Surgical outcome data reveal a high satisfaction rate with this procedure, but it continues to have a significant complication rate, often requiring revision and/or explantation. In addition, there is a lack of standardisation in current reporting of AUS outcomes. Overall, literature on the surgical treatment of male SUI requires standardised reporting of workup and outcome measures, as well as complete collation and reporting of adverse events and long-term results.^6,7^ Guidelines on post-prostatectomy incontinence (PPI) also have variations, even though they might share overarching principles.^8^ Several consensus documents have been published regarding the indications, management and follow-up of AMS800 implantation or revision.^9–11^

Considering the increased use of AUS and the lack of reported standardisation in AUS practice, as well as male SUI surgery, the aim of this study was to understand the current practice of AUS insertion in the UK using a British Association of Urological Surgeons (BAUS)-approved survey and to compare these data with the practice standards from guideline documents.

## Methods

A survey of current practice of AUS surgery in the UK was performed. The Checklist for Reporting Results of Internet E-Surveys survey was followed for reporting the survey results.^12^

### Design

The survey targeted UK-based urologists performing male SUI surgery, and a combination of sampling techniques were used. The survey was endorsed by the BAUS Section of Female, Neurological and Urodynamic Urology (FNUU) and sent to email addresses on their mailing list (probability list-based sampling). To increase the response rate, this was combined with a non-probability sampling technique consisting of an unrestricted self-selected survey method by including a link on social media platforms such as X (formerly Twitter).

The survey did not require ethical approval, and participation in the survey was considered to be consent from the survey participants. The survey was anonymous, and responses were stored in a password-protected REDCap database. Responses were accessible only by the first author. A copy of the survey questions is available online in Appendix 1.

### Development and pretesting

The REDCap survey was piloted by the authors prior to circulation. The scope, choice of questions and format were discussed at length by the author group, which consisted of several members of the BAUS FNUU as well as UK urologists performing male SUI surgery. The formatting, sense, reliability, ease of use and functionality of the survey were tested by the author group.

### Recruitment process

The survey was advertised in January 2024 and was open for 4 months from 11 January to 11 May 2024. The sampling frames above were used for advertisement. Urologists working in the UK performing male SUI surgery could participate in the survey, which aimed to collect data on their male SUI surgery practice.

### Preventing multiple entries

The survey collected anonymised information on the respondent’s area of practice, experience and timing of survey completion, which allowed us to find and remove any duplicate records.

### Survey content

The survey consisted of a combination of closed and open-ended questions along with free text to allow respondents to explain or elaborate on their choices. The themes explored included: surgeon characteristics, preoperative workup, surgical options, surgical approach, intraoperative steps, postoperative plan and special cases or complication management.

### Outcomes

The outcomes for the survey study were framed using the International Continence Society (ICS) consensus document on AUS, which has eight chapters, and the recent BAUS consensus document.^10,11^ The BAUS consensus was published soon after the survey was conducted, but a comparison to current UK practice is made to understand areas the need for more standardisation if any.

### Primary and secondary outcomes

The primary outcome of the study was to determine the presence of any variability in the current practice surrounding AUS surgery in the UK. Secondary outcomes were to:

• determine the preoperative assessment and challenges in patients undergoing AUS surgery in the UK;
• determine the implantation technique used by urologists performing AUS surgery in the UK;
• elucidate the postoperative care of patients undergoing AUS surgery across the UK;
• understand the management of complications (troubleshooting) or approach in special populations of AUS insertion across the UK.

### Analysis

The overall response rate was determined based on fully completed questionnaires. Partially completed questionnaire data were used in terms of the questions that were answered; these were included in the analysis. Descriptive statistics were performed after analysing the data for normality.

## Results

### Response rates

The sampling frame using the mailing list consisted of 1,337 members of BAUS; however, the number of surgeons across the UK performing male SUI surgery is likely to be much lower. It is currently not possible to determine exactly how many surgeons across the UK are performing this operation. The Tweet generated 3,411 views, 205 engagements and 3,400 views.

Thirty-six surgeons replied to the survey, of whom 34 were eligible to answer; 2 respondents were not practising consultants in the UK, and so were ineligible. Because not all the questions were mandatory, the response rate per question varied from 44% to 94% across the domains.

### Surgeon characteristics

**Figure 2:**
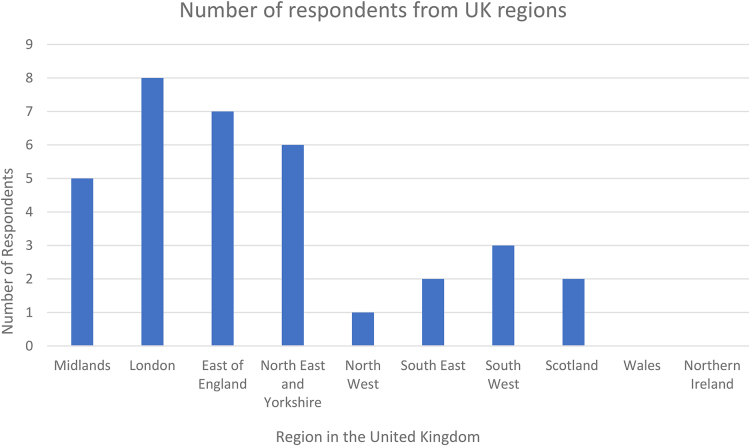
Number of respondents per region of the UK

**Figure 3:**
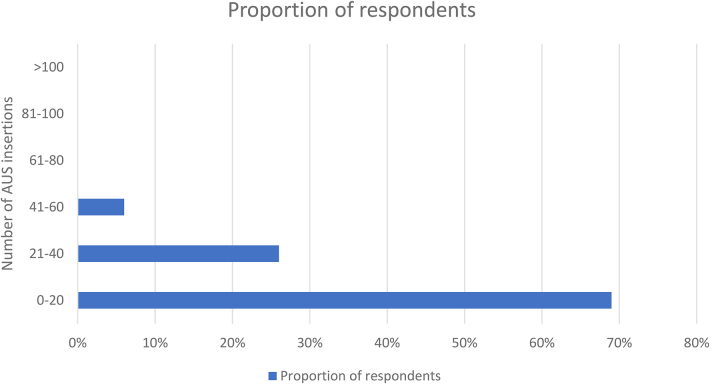
Number of artificial urinary sphincter insertions performed by each surgeon in the UK as per the survey

Respondents included consultant urological surgeons from different regions of the country (Figure 2). Surgeon experience was varied, ranging from less than 5 years (*n* = 9, 29%) to more than 15 years (*n* = 5, 16%). More than two-thirds of the surgeons (*n* = 23, 70%) performed 20 or fewer AUS insertions a year, and only 2 respondents performed 41–60 AUS insertions a year (Figure 3). A prostate cancer survivorship programme is currently available in 63% (*n* = 19) of respondent’s centres.

### Preoperative assessment

#### Investigations

Investigations performed routinely prior to AUS insertion included: video-urodynamics (*n* = 27, 85%); patient-reported outcome measures (PROMs), e.g. the International Consultation on Incontinence Questionnaires (*n* = 15, 78%); flexible cystourethroscopy (*n* = 21, 66%); pad test, i.e. number and pad weight measurement (*n* = 15, 47%); standard urodynamics (*n* = 5, 16%); and imaging, e.g. upper tract or magnetic resonance imaging scan (*n* = 1, 3%).

Some of the urodynamics findings would prompt clinicians to not offer the sphincter or offer it only with counselling; these were: loss of compliance (*n* = 26, 93%); physiological bladder capacity <200ml (*n* = 19, 68%); detrusor overactivity (DO) of any type (*n* = 13, 46%); and detrusor underactivity during voiding (*n* = 9, 32%).

The approach to patients with DO on the urodynamics was variable, but most respondents attempt to treat the DO first and then offer SUI surgery (in 3–6 months) even if the patient may not respond to treatment. Some respondents offer appropriate counselling regarding the need for further treatment (refractory DO) (*n* = 24, 73%), and the remainder treat the DO and offer surgery only if the DO responds regardless of duration (*n* = 4, 12%) or do not offer surgery in patients with preoperative DO (*n* = 5, 15%).

#### Severity classification

All respondents use some form of severity classification for male incontinence; however, the type of classification used varied. The most commonly used classification was pad weight according to the MASTER trial^13^: <200 to 250g per 24h was considered mild to moderate incontinence and >200 to 250g per 24h was considered severe incontinence (*n* = 6, 40%).

The other commonly used severity measures included the Nitti classification: pad weight <100g (mild), 100–400g (moderate), >400g (severe) (*n* = 4, 27%); pad number per 24h, <2 (mild), 2–5 (moderate) and 5 (severe) (*n* = 2, 13%). Some clinicians did not use a strict weight or number criteria, but preferred using estimates (*n* = 4, 27%).

#### Surgical options offered

AUS was the most commonly offered male SUI operation for all respondents, followed by male sling locally (*n* = 16, 50%); six respondents referred their patients to other centres for the sling and only six respondents offered bulking agents to this cohort. More than half the respondents offered all options to the patient regardless of severity (*n* = 16, 62%), and nearly one-third (10, 38%) offered all options for mild–moderate incontinence, but AUS only for severe incontinence.

#### Radiotherapy

In patients with incontinence post-prostatectomy who were awaiting radiotherapy, almost 65% of respondents would offer male incontinence surgery prior to salvage radiotherapy. For patients who had undergone previous radiotherapy, more than half of the respondents (*n* = 14, 52%) would offer only AUS and not the sling with counselling. The remaining respondents offered all options while counselling patients about the poor overall outcomes (*n* = 5, 18%) or poor outcomes just with the sling (*n* = 6, 22%).

In the case of previous radiotherapy, most clinicians preferred waiting 6–12 months before offering male SUI surgery (*n* = 18, 64%). The remaining responses were split between not waiting a fixed period (*n* = 5, 18%) and waiting 1–2 years after radiotherapy (*n* = 3, 11%).

#### Surgical approach: preoperative planning

The approach to a positive midstream specimen of urine preoperatively was divided between treating the infection and continuing with the operative plan as before (*n* = 16, 48%) or postponing the surgery until a negative sample was obtained (*n* = 15, 45%). Some clinicians (*n* = 2, 6%) would start patients on low-dose antibiotics after treating the infection until the procedure.

Nearly all clinicians give patients broad-spectrum antibiotics at induction of AUS surgery (*n* = 28, 87%) and half give intravenous antibiotics for 24h postoperatively (*n* = 16, 50%). Oral antibiotics were given for 5 days (*n* = 7, 22%) or 7 days (*n* = 5, 16%) postoperatively. The most commonly used antibiotics were penicillin-based, e.g. co-amoxiclav, in patients who were not allergic to them.

Preoperative preparation for AUS surgery was highly variable, the approaches used included: 10-min scrub preoperatively on table (*n* = 11, 35%); routine preparation and drape only (*n* = 10, 31%); 5-min scrub preoperatively on table (*n* = 13, 41%); and preoperative body wash/disinfectant agent for patient (*n* = 5, 16%).

The most common agent used for preoperative preparation was 4% undiluted chlorhexidine (*n* = 13, 41%). Other agents used according to preference are: betadine 7.5% diluted (*n* = 3, 9%); betadine 10% diluted (*n* = 5, 16%); chlorhexidine 4% diluted (*n* = 7, 22%); betadine 7.5% undiluted (*n* = 4, 12%); betadine 10% undiluted (*n* = 3, 9.4%); alcoholic betadine (*n* = 2, 6%); and aqueous betadine (*n* = 1, 3%).

### Implantation technique

#### Catheter care

All clinicians use a catheter intraoperatively, most leave one in place postoperatively (*n* = 25, 76%) and only eight respondents remove this after the surgery (24%). The most common catheter size used for this purpose was 14Fr (*n* = 14, 42%), followed by 16Fr (*n* = 13, 39%) and 12Fr (*n* = 6, 18%).

#### Steps performed

In terms of intraoperative steps, most clinicians (*n* = 29, 91%) use implant precautions such as minimal staff in theatre, laminar air flow and locking doors. It is common practice to always use antibiotic-impregnated devices (*n* = 27, 84%), use antibiotic wash on table, e.g. rifampicin (*n* = 23, 72%), and measure cuff size with the catheter in situ (*n* = 26, 81%). Other steps performed less commonly include rotating the cuff to the dorsal aspect of the urethra (*n* = 17, 53%), cystourethroscopy on the table before incision (*n* = 9, 28%) and removing the catheter prior to cuff sizing (*n* = 5, 16%).

#### Cuff

The 3.5cm cuff is used less commonly in the UK, with more than two-thirds of respondents not using it routinely or never having used it (*n* = 20, 68%). A small number of clinicians use it for revision cases only (*n* = 6, 21%), whereas only three respondents in the UK (10%) routinely use this cuff.

#### Reservoir placement

All respondents use the 61–70cmH_2_O reservoir routinely, although some consider using the 71–80cmH_2_O depending on the situation (*n* = 9, 28%). The preferred location of the reservoir was variable, including: extraperitoneal only (*n* = 11, 34%); retropubic only (*n* = 7, 22%); and either location depending on the patient (*n* = 14, 44%). The most common type of reservoir used by respondents was saline (*n* = 19, 60%) with some using contrast or both depending on allergy status or availability (*n* = 8, 25%).

#### Approach to pump placement

With regards to placement, the pump is most commonly placed on the dominant side of the patient (*n* = 24, 77%) and more than half the respondents instruct patients to massage the pump down several times a day (*n* = 16, 52%). Some respondents place the pump either side regardless of the dominant side (*n* = 10, 32%) and instruct patients to not touch the pump after placement (*n* = 8, 26%). A very small proportion (*n* = 2, 7%) suture the superficial ring to prevent pump migration.

### Postoperative care

#### Postoperative length of stay

The AUS procedure is most commonly performed as a 23h stay by urologists in the UK (*n* = 25, 76%); a small number perform this as a day case procedure without overnight stay (*n* = 5, 15%) or >24h stay (*n* = 3, 9%). All respondents see patients 4–6 weeks after surgery for activation.

#### Postoperative follow-up

After 4–6 weeks, patient follow-up, as per the responses, was variable and included follow-up at 3 months (*n* = 14, 44%), 6 months (*n* = 5, 16%) and 12 months (*n* = 8, 25%). In terms of longer-term follow-up, patients were most commonly placed on patient-initiated follow-up for a fixed period ( *n* = 15, 47%); a small proportion of respondents see patients annually (*n* = 5, 16%) or do not arrange routine follow-up with an option to contact the specialist nurse or surgeon’s secretary in case of any issues (*n* = 7, 22%).

At the follow-up appointment, the most commonly collected data included PROMs (*n* = 18, 56%). More than one-quarter of the respondents do not collect any routine data if the patient has no complaints. Other parameters collected included: history and exam (*n* = 10, 31%); cough stress test (*n* = 3, 9%); pad weight and pad use (*n* = 5, 16%); and uroflow with post-void residual (*n* = 1, 3%).

### Management of complications (troubleshooting/special populations)

#### Retention

In the case of urinary retention after catheter removal in the early postoperative period, most respondents would reinsert a 12Fr or 14Fr catheter and perform a trial of void without catheter (TWOC) within 24–48h (*n* = 22, 67%); the remaining respondents do so at >48h (*n* = 11, 33%). If the patient were to go into retention following this, the approach used by respondents was variable and included: reinsertion of a urethral catheter and TWOC within 1–2 weeks (*n* = 13, 39%); intermittent catheterisation (*n* = 15, 45%); insertion of a suprapubic catheter (*n* = 9, 27%); and revision of the device (*n* = 1, 3%).

#### Bladder neck stenosis

In case of bladder neck stenosis or urethral stricture, fewer than half the respondents wait 6 months (*n* = 15, 46%) before offering an AUS; the remaining respondents wait either 3 months (*n* = 11, 33%) or 12 months (*n* = 7, 21%).

#### Mechanical failure

In case of mechanical failure of the AUS device, the most common approach is to replace the entire device (*n* = 19, 58%); the remaining respondents make a decision based on the time since insertion, i.e. replace the components if the device was inserted <2 years ago or replace the entire device if inserted >2 years ago (*n* = 13, 39%).

## Discussion

This national survey provides an up-to-date insight into the current landscape of AUS surgery in the UK. The survey highlights significant variation in preoperative assessment, intraoperative technique and postoperative management, reflecting a broader pattern on variability and inconsistency reported in benign urological surgery in general. AUS remains the gold standard treatment for male incontinence surgery; however, our study reinforces the need for standardisation and adherence to UK and international guidelines, especially as the number of procedures performed continues to grow steadily.^11^^,12,14–19^

The survey findings revealed that AUS surgery is available across all seven NHS England regions and in Scotland, but our results align with international trends reporting most surgeons perform this procedure infrequently: two-thirds of UK respondents perform 20 or fewer insertions per year. Low-volume practice raises concerns about surgical proficiency maintenance and optimal patient outcomes, as reflected in the literature linking surgeon experience to AUS revision and failure rates. Our survey shows UK centres are progressively integrating prostate cancer survivorship into clinical practice in line with the recent BAUS consensus.^11^

Surgical options for PPI include AUS and the male sling, but the male sling is offered less commonly than AUS in the UK. The suspension of mesh anti-incontinence surgery in women using vaginally inserted mesh may result in bias in the surgical options offered and chosen by patients in the UK, compared with practice in other parts of the world.^20^ Our findings underscore major variability in preoperative workup. Although most centres routinely perform video-urodynamics, wide variation was reported in the use of other investigations such as pad weight testing, PROMs, cystoscopy and standard urodynamics. In addition, the thresholds for excluding or counselling patients based on urodynamics (e.g. DO or low compliance) were not consistent. This heterogeneity most likely stems from the absence of a unified clinical pathway for evaluating male incontinence internationally, resulting in inconsistent patient selection and counselling. The new BAUS guidelines provide a consensus pathway for the workup of these patients, which might result in a reduction in the current heterogeneity in practice.

Variation was noted in perioperative planning, including the approach to infection control, antibiotic regimens and surgical preparation (e.g. preoperative scrub and antiseptic agents). Infection control measures are adopted by most urologists, but variability in practice mirrors that observed in penile prosthetic surgery.^21^ Our knowledge concerning the prevention of prosthetic infections largely stems from extrapolations made from the orthopaedic and general surgery literature. Consequently, this has resulted in notable discrepancies in surgical preparation methods and techniques. The absence of randomised clinical trials directly comparing preoperative antibiotics and preparation procedures is glaring, and the prospects of such trials being conducted are slim. Thus, at present, consensus documents are our most reliable source for guiding clinical practice in this domain.

Key technical aspects of AUS implantation, e.g. cuff sizing, reservoir pressure selection and pump positioning, also showed variation. The recent BAUS and ICS guidelines are the only ones providing detail on the perioperative care of AUS patients.^11^ A comparison of the available implantation techniques in the ICS consensus with the findings from this survey is given in Table 1. Postoperative follow-up pathways further exemplify this variability. Although most surgeons reviewed patients at 4–6 weeks for device activation, subsequent follow-up protocols varied, ranging from annual visits to patient-initiated contact only. Data collection during follow-up was similarly inconsistent, with only 56% of respondents collecting PROMs and very few capturing objective data such as pad weights or uroflow metrics. This lack of structured follow-up limits the ability to benchmark outcomes and audit complications, both of which are crucial for quality assurance in benign surgery. A 23-h postoperative stay is most common with activation at 4–6 weeks. However, day case AUS implantation is also being practised in the UK in the face of emerging evidence of its suitability.^22,23^

**Table 1:** Comparison of perioperative artificial urinary sphincter practice responses in the UK with the British Association of Urological Surgeons (BAUS) and International Continence Society (ICS) consensus

Perioperative care step	ICS and BAUS consensus recommendation	Current survey results (most common response)
Preoperative scrub	5-min scrub, chlorhexidine superior to povidone–iodine	10-min scrub with 4% undiluted chlorhexidine
Postoperative antibiotics	No evidence to support practice	24h intravenous antibiotics by half the respondents Further oral antibiotics for 5–7 days by more than one-third
Catheter use	14Fr or less catheter size overnight, not over 48h	14Fr catheter size used by fewer than half the respondents (42%)
3.5cm cuff	Can be used in irradiated patients	More than half the respondents do not use this routinely or never have used it
Antibiotic-coated AUS	Do not reduce infection rates, are costlier	Preferred by 84% respondents in the UK
Reservoir size	61–70cmH_2_O	61–70cmH_2_O Placement in abdomen variable, saline reservoir preferred as per responses
Pump placement	Pull down on the pump several times a day and typically placing it on the ipsilateral dominant side	More than half the respondents place follow this practice in the UK
Long-term follow-up	Follow-up at 3 and 6 months postoperatively and periodically after, and further annual follow-up in person or by questionnaire with mandatory evaluation of symptoms consistent with device malfunction, infection and/or erosion	The most common approach for longer-term follow-up in the UK is a patient-initiated follow-up pathway as per half the respondents. One-third of the respondents do not collect any follow-up data even if they review the patients longer term

Complication management and complex scenarios, e.g. patients with previous radiotherapy or bladder neck stenosis, and revision surgery, was variable, showing no dominant practice pattern and reflecting a lack of consensus-driven protocols. This might change with the BAUS consensus document, which offers a detailed overview of managing these scenarios.

Systematic reviews reporting AUS outcomes found that the evidence in this area was limited by significant variations in the evaluation of outcomes of PPI surgery, definition of continence achieved by surgery and the inconsistent follow-up duration. Also reported was a difference in surgical methods between different surgeons, such as the timing of implantation, incision design and choice of suture methods, as seen in our survey.^24^ Overall, the quality of evidence is low, based on heterogeneous data. This is evident from the literature in the wide confidence intervals for the complications reported, e.g. infection, erosion. Hence, such variation can potentially have an impact on not only the surgical outcomes, but also reporting of the outcomes and research in this area.^25^ These findings speak to a broader issue in benign surgery of increased variability in practice despite guidelines and consensus documents. This can be attributed in part to historically lower prioritisation of benign conditions compared with oncological conditions, leading to reduced standardisation, lower research funding and less-robust training pathways. The COVID-19 pandemic further exacerbated this disparity by disproportionately affecting elective benign surgery such as AUS insertion.

Variation in surgical care has been recognised since the early 1980s and is often seen as a reflection of uncertainty among medical professionals regarding optimal care.^26^ Variability can have a negative impact, resulting in a loss of value in the care we provide to our patients because important variations in surgical practice consequently lead to important variations in the outcomes.^24–26^ Those who use and commission surgical services want to be confident of receiving consistent high-quality care based on best available evidence.^24–26^ Surgical rates for a condition that vary independent of the disease prevalence may suggest that surgery might be done without regard to equity or evidence. The greatest variable in this context appears to be surgeon attitudes. Solutions to reducing variation include the development of better evidence, and the implementation of clinical practice guidelines and care pathways to streamline care. In line with this, we hope to see a reduction in the variability of surgical practice in UK with the recent publication of the BAUS consensus tailored to the UK context.^11^ Standardisation of the practice highlighted in this consensus could be fed into curriculum development for AUS surgery to further ensure reduction in variability and uniformity in training for this surgery. In addition, national databases, mandatory outcome reporting and surgeon accreditation may be necessary to align real-world practice with evidence-based standards. The creation of centres of excellence or regionalised care models for AUS surgery could also help consolidate expertise and improve outcomes. Furthermore, future work should examine patient perspectives and outcomes considering these practice variations, particularly in lower-volume centres.

### Study limitations

This survey has limitations in terms of the small cohort of respondents, but this could be an approximate reflection of the number of clinicians implanting AUS in the UK. The survey responses are used as a proxy for the clinicians’ actual practices, representing a limitation of this methodology.

## Conclusions

In summary, this national survey confirms significant variability in the practice of AUS surgery across the UK from preoperative assessment to long-term follow-up, together with notable deviations from international guidelines, including the recently published BAUS consensus. These differences may stem from local or national influences, a lack of high-quality evidence and individuals’ surgical training, which may diverge from established guidelines and consensus standards. The effect of the current variability in AUS surgery can be seen in the heterogeneity of outcomes and reporting of such outcomes. Considering these findings, we hope that the recently published BAUS consensus will standardise practice, and other measures such as curriculum development to standardise training, research into effect of variability on surgical outcomes and an overall effort to improve the quality of evidence through audit and regional collaboration will be helpful to reduce variability.

## Competing interests

HH: Medtronic – trial investigator, educational grant, speaker, mentor; Astellas – speaker, advisory board; Boston Scientific – trial investigator, mentor; Abbvie – speaker. AS: Boston Scientific – educational grant, speaker; Abbvie – speaker fees, conference/travel fees; Medtronic – trial investigator, educational grant, speaker fees. SB: education and training for Boston Scientific.

## Funding

The author/s received no financial support for the research, authorship and/or publication of this article.

## Ethics approval and consent to participate

Not applicable.

## Author contributions

**NR Bhatt**: Conceptualisation, Data curation, Formal analysis, Investigation, Methodology, Resources, Software, Writing – original draft. **R Doherty**: Conceptualisation, Supervision, Writing – review & editing. **S Biers**: Writing – review & editing. **C Harding**: Supervision, Writing – review & editing. **N Thiruchelvam**: Supervision, Writing – review & editing. **M Belal**: Supervision, Writing – review & editing. **A Sahai**: Supervision, Writing – review & editing. **H Hashim**: Conceptualisation, Supervision, Writing – review & editing.

## Artificial Intelligence

The author/s declare that no AI was used to conduct the study or prepare the manuscript.

## Supplementary Information

The online version contains supplementary material available at https://doi.org/10.1308/rcsann.2025.0104.

## References

[1] Islah M, Cho SY, Son H. The current role of the artificial urinary sphincter in male and female urinary incontinence. *World J Mens Health* 2013; **31**: 21–30. 23658862 10.5534/wjmh.2013.31.1.21 PMC3640149

[2] Cordon BH, Singla N, Singla AK. Artificial urinary sphincters for male stress urinary incontinence: current perspectives. *Med Devices (Auckl)* 2016; **9**: 175–183. 27445509 10.2147/MDER.S93637 PMC4938139

[3] Department of Health, UK. Hospital Episode Statistics. http://www.hscic.gov.uk/hes (cited April 2023).

[4] Lee R, Te AE, Kaplan SA, Sandhu JS. Temporal trends in adoption of and indications for the artificial urinary sphincter. *J Urol* 2009; **181**: 2622–2627. 19375102 10.1016/j.juro.2009.01.113

[5] Ratan HL, Summerton DJ, Wilson SK, Terry TR. Development and current status of the AMS 800 artificial urinary sphincter. *EAU-EBU Update Ser* 2006; **4**: 117–128.

[6] Montague DK. Artificial urinary sphincter: long-term results and patient satisfaction. *Adv Urol* 2012; **2012**: 835290. 22536227 10.1155/2012/835290 PMC3318201

[7] Herschorn S, Bruschini H, Comiter C *et al.* Surgical treatment of stress incontinence in men. *Neurourol Urodyn* 2010; **29**: 179–190. 20025026 10.1002/nau.20844

[8] Bhatt NR, Pavithran A, Ilie C *et al.* Post-prostatectomy incontinence: a guideline of guidelines. *BJU Int* 2024; **133**: 513–523. 38009420 10.1111/bju.16233

[9] Chung E, Liao L, Kim JH *et al.* The Asia-Pacific AMS800 artificial urinary sphincter consensus statement. *Int J Urol* 2023; **30**: 128–138. 36375037 10.1111/iju.15083 PMC10100264

[10] Biardeau X, Aharony S, Campeau L, Corcos J. Artificial urinary sphincter: report of the 2015 consensus conference. *Neurourol Urodyn* 2016; **35**: S8–24. 27064055 10.1002/nau.22989

[11] Eysenbach G. Improving the quality of web surveys: the Checklist for Reporting Results of Internet E-surveys (CHERRIES). *J Med Internet Res* 2004; **6**: e34. 15471760 10.2196/jmir.6.3.e34 PMC1550605

[12] Abrams P, Constable LD, Cooper D *et al.* Outcomes of a noninferiority randomised controlled trial of surgery for men with urodynamic stress incontinence after prostate surgery (MASTER). *Eur Urol* 2021; **79**: 812–823. 33551297 10.1016/j.eururo.2021.01.024 PMC8175331

[13] Sandhu JS, Breyer B, Comiter C *et al.* Incontinence after prostate treatment: AUA/SUFU guideline. *J Urol* 2019; **202**: 369–378. 31059663 10.1097/JU.0000000000000314

[14] Gacci M, Sakalis VI, Karavitakis M *et al.* European association of urology guidelines on male urinary incontinence. *Eur Urol* 2022; **82**: 387–398. 35697561 10.1016/j.eururo.2022.05.012

[15] Bettez M, Tu LM, Carlson K *et al.* 2012 update: guidelines for adult urinary incontinence collaborative consensus document for the Canadian urological association. *Can Urol Assoc J; J l’Association des Urol du Canada* 2012; **6**: 354–363. 10.5489/cuaj.12248 PMC3478335 23093627

[16] Sinha S, Agarwal MM, Vasudeva P *et al.* The urological society of India guidelines for the evaluation and management of nonneurogenic urinary incontinence in adults (executive summary). *Indian J Urol* 2019; **35**: 185–188. 31367068 10.4103/iju.IJU_125_19 PMC6639992

[17] Herschorn S, Bruschini H, Comiter C *et al.* Surgical treatment of urinary incontinence in men. In: ICS. 2023. pp1121–1190.

[18] Averbeck MA, Woodhouse C, Comiter C *et al.* Surgical treatment of post-prostatectomy stress urinary incontinence in adult men: report from the 6th international consultation on incontinence. *Neurourol Urodyn* 2019; **38**: 398–406. 30350875 10.1002/nau.23845

[19] Wise J. Surgical mesh for stress urinary incontinence to be halted immediately in England. *BMJ* 2018; **362**: k3035. 29991515 10.1136/bmj.k3035

[20] Masterson TA, Palmer J, Dubin J, Ramasamy R. Medical pre-operative considerations for patients undergoing penile implantation. *Transl Androl Urol* 2017; **6**: S824–S829. 29238662 10.21037/tau.2017.03.85 PMC5715179

[21] Dropkin BM, Sanders SC, Kavoussi M *et al.* Same day discharge versus overnight observation protocols – similar outcomes following artificial urinary sphincter surgery. *Urology* 2021; **157**: 206–210. 34437897 10.1016/j.urology.2021.08.016

[22] Myrga JM, Vasan R, Miller DT *et al.* Catheter free day of surgery discharge vs overnight observation following artificial urinary sphincter placement. *Cureus* 2023; **15**: e36898. 37128518 10.7759/cureus.36898 PMC10148565

[23] Li Y, Li X, Yang Q. Effectiveness of artificial urinary sphincter to treat stress incontinence after prostatectomy: A meta-analysis and systematic review. *PLoS ONE* 2023; **18**: e0290949. 37656677 10.1371/journal.pone.0290949 PMC10473540

[24] Urbach DR, Baxter NN. Reducing variation in surgical care. *BMJ* 2005; **330**: 1401–1402. 15961794 10.1136/bmj.330.7505.1401 PMC558361

[25] Pera M. Variability in surgical practice. An unresolved problem. *Cir Esp* 2017; **95**: 59–61. 28274331 10.1016/j.ciresp.2017.02.006

[26] Variation in surgery and surgical research. *Lancet* 2013; **382**: 1071. 24075031 10.1016/S0140-6736(13)62006-1

